# The anesthetist’s choice of inhalational vs. intravenous anesthetics has no impact on survival of glioblastoma patients

**DOI:** 10.1007/s10143-020-01452-7

**Published:** 2020-12-22

**Authors:** Thomas Schmoch, Christine Jungk, Thomas Bruckner, Sabine Haag, Klaus Zweckberger, Andreas von Deimling, Thorsten Brenner, Andreas Unterberg, Markus A. Weigand, Florian Uhle, Christel Herold-Mende

**Affiliations:** 1 Department of Anesthesiology and Intensive Care Medicine, University Hospital Essen, Hufelandstraße 55, 45147 Essen, Germany; 2 Department of Anesthesiology, University Hospital Heidelberg, Heidelberg, Germany; 3 Division of Neurosurgical Research, Department of Neurosurgery, University Hospital Heidelberg, Heidelberg, Germany; 4 Institute of Medical Biometry and Informatics (IMBI), University of Heidelberg, Heidelberg, Germany; 5 Department of Neuropathology, University Hospital Heidelberg and Clinical Cooperation Unit (CCU) Neuropathology, DKFZ, Heidelberg, Germany

**Keywords:** TIVA, Inhalational anesthesia, Glioblastoma, Aurvival

## Abstract

Recent data suggest that the type of anesthesia used during the resection of solid tumors impacts the long-term survival of patients favoring total-intravenous-anesthesia (TIVA) over inhalative-anesthesia (INHA). Here we sought to query this impact on survival in patients undergoing resection of glioblastoma (GBM). All patients receiving elective resection of a newly diagnosed, isocitrate-dehydrogenase-1-(IDH1)-wildtype GBM under general anesthesia between January 2010 and June 2017 in the Department of Neurosurgery, Heidelberg University Hospital, were included. Patients were grouped according to the applied anesthetic technique. To adjust for potential prognostic confounders, patients were matched in a 1:2 ratio (TIVA vs. INHA), taking into account the known prognostic factors: age, extent of resection, O-6-methylguanine-DNA-methyltransferase-(MGMT)-promoter-methylation-status, pre-operative Karnofsky-performance-index and adjuvant radio- and chemotherapy. The primary endpoint was progression-free-survival (PFS) and the secondary endpoint was overall-survival (OS). In the study period, 576 patients underwent resection of a newly diagnosed, IDH-wildtype GBM. Patients with incomplete follow-up-data, on palliative treatment, having emergency or awake surgery; 54 patients remained in the TIVA-group and 417 in the INHA-group. After matching, 52 patients remained in the TIVA-group and 92 in the INHA-group. Median PFS was 6 months in both groups. The median OS was 13.5 months in the TIVA-group and 13.0 months in the INHA-group. No significant survival differences associated with the type of anesthesia were found either before or after adjustment for known prognostic factors. This retrospective study supports the notion that the current anesthetic approaches employed during the resection of IDH-wildtype GBM do not impact patient survival.

**Supplementary Information:**

The online version contains supplementary material available at 10.1007/s10143-020-01452-7.

## Introduction

High-grade gliomas are the most frequent and aggressive primary brain tumors in adults, with glioblastoma (GBM) being the most common among them [13]. The WHO distinguishes “IDH-mutant“ GBM, harboring a mutation in the isocitrate-dehydrogenase-1-(IDH1)-gene, from “IDH-wild type” GBM (~95% of cases) [13, 17]. IDH-mutant GBMs are associated with a significantly longer overall-survival [16]. Moreover, age at time of surgery, extent of resection (EOR), and pre-operative Karnofsky-performance-index (KPI) are known prognostic factors [21, 23]. Additionally, the promoter-methylation-status of the gene coding O-6-methylguanine-DNA-methyltransferase (MGMT) predicts effectiveness of alkylating chemotherapy. Standard therapy consists of surgical resection followed by radiotherapy combined with adjuvant chemotherapy using the alkylating agent temozolomide (TMZ) [1, 5, 12, 15].

One important factor influencing the long-term outcome of patients suffering from high-grade gliomas might have been neglected so far, in that resection of the main tumor mass is usually performed under general anesthesia (exceptions are cases of awake surgery). The hypothesis that the hypnotic agent used during resection influences dissemination of tumor cells into the blood circulation or cerebrospinal fluid has been increasingly supported recently [8]. Wigmore et al. retrospectively analyzed medical histories of 3070 patients with solid tumors of different entities who underwent tumor resection under general anesthesia maintained either as inhalational-anesthesia (INHA) or total-intravenous-anesthesia (TIVA) [26]. Within the observation period of 4 years, mortality in the INHA group was 24 versus 13.5% in the TIVA group. The difference remained significant after propensity matching. Although these results were not adjusted for tumor entities and their specific prognostic factors, similar results were found for colon [4] and breast cancer [10].

So far, influence of the employed anesthetic technique (AT) on survival of GBM patients has been investigated in only one retrospective cohort [3] with limited informative value as the observation time was relatively short (10 months) and groups were not adjusted for known prognostic factors. However, data of Wigmore et al. [26] strongly recommend analyzing the influence of TIVA and INHA in this particular tumor type, as there is growing evidence from experimental studies that the intravenous anesthetic agent propofol [27, 28] might have a more favorable effect on proliferation and invasiveness of glioma cells than the volatile anesthetic “sevoflurane” [22]. Presently, both INHA and TIVA are widely accepted for anesthetic management of supratentorial intracranial surgery [9, 14, 18, 20]. Consequently, at our institution, both ATs are used according to the anesthetist’s preference. Therefore, we retrospectively reviewed the outcome of patients undergoing resection of newly diagnosed IDH-wildtype GBM dependent on the employed AT.

## Materials and methods

### Study design

This retrospective cohort study was approved by the Institutional-Review-Board (IRB, Votum S-843/2018 (Medical Ethics Commission of the Medical Faculty of Heidelberg University, Heidelberg, Germany**)** and conducted in accordance with ethical standards of the latest version of the Helsinki Declaration (July 9, 2018) [24]. Requirement for written informed consent was waived by the IRB. This manuscript adheres to the applicable guidelines of the Enhancing the Quality of and Transparency of Health Research (EQUATOR) Network.

### Participants

**Fig. 1:**
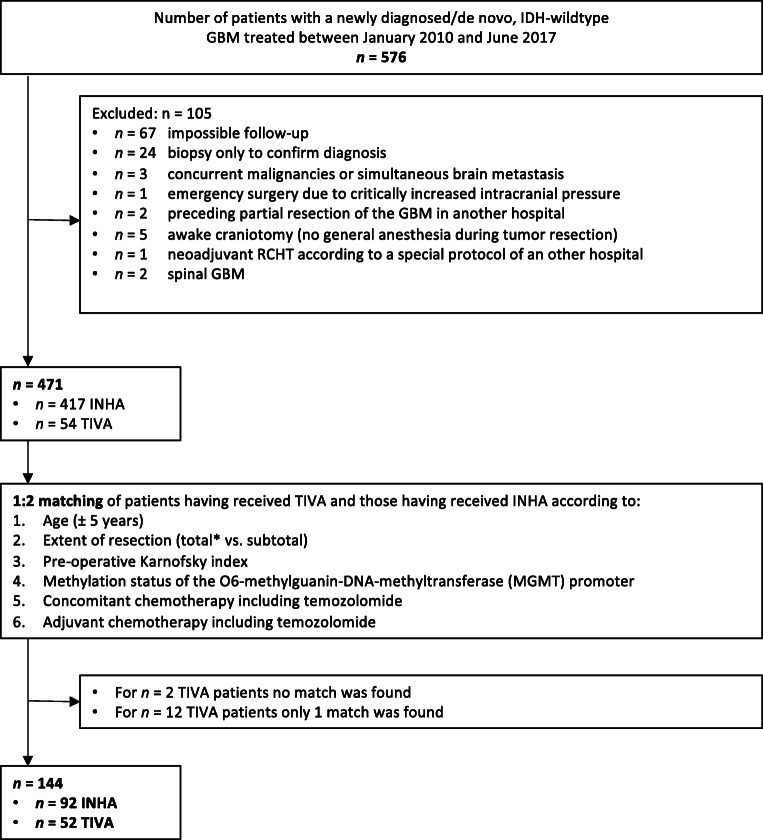
Flow diagram detailing the selection of patients included in the retrospective analysis. Patients who had further procedures during the study period remained eligible regardless of the anesthetic technique (AT), as we were interested in the effects on resection of the main tumor mass. *Gross total resection was defined as no residual nodular contrast enhancement on early post-operative MRI within 24–72 h post-surgery. *GBM* glioblastoma multiforme, *IDH* isocitrate-dehydrogenase 1, *INHA* volatile inhalational anesthesia, *MRI* magnetic resonance imaging, *TIVA* total intravenous anesthesia, *RCHT* radio- and chemotherapy

All adult patients (≥ 18 years) undergoing resection of a newly diagnosed, IDH-wildtype GBM under general anesthesia between January 1, 2010, and June 30, 2017, at the Department of Neurosurgery, University Hospital Heidelberg (Germany), were included (*n* = 576). Exclusion criteria were biopsy cases, incomplete outcome-data, palliative treatment after surgery, simultaneous treatment of other malignancies, emergency or awake surgery, spinal tumor location, neoadjuvant radio-chemotherapy (before resection), and switch of the type of anesthesia (TIVA or INHA) during tumor resection. A total of 471 patients met inclusion criteria (Fig. 1). Patients were grouped according to whether they had received INHA (*n* = 417) or TIVA (*n* = 54). Patients had received continuous infusions of propofol in the TIVA group and the volatile inhalational agent sevoflurane in the INHA group. Type of anesthesia was chosen according to the anesthetist’s preference. Patients with critically increased intracranial pressure requiring emergency surgery under TIVA were excluded to avoid potential bias. In 16/54 cases (33.3%), TIVA was chosen due to intraoperative neurophysiological monitoring (IONM). Patients in both groups received sufentanil or remifentanil as a supplementary opioid (anesthesiologist’s preference). No patient received nitrous oxide. In all patients, general anesthesia was started using a single dose of propofol (2 mg/kg), the opioid “sufentanil” and the muscle relaxant “rocuronium.” We did not take into account the type of anesthesia for additional procedures because we sought to evaluate the impact of the type of anesthesia during resection of the main tumor mass.

### Variables

Patient data included AT, IDH status, age at time of surgery, gender, EOR, MGMT-promoter-methylation-status, pre-operative KPI, concomitant and adjuvant radiation and chemotherapy, intra-operative blood transfusion, body mass index (BMI), use of opioids and relaxants, progression-free survival (PFS), and OS. The use of opioids and relaxants was not included in the analysis as all cases received them. Pre-operative morbidity was assessed by the American Society of Anesthesiologists (ASA) rating scale (as pre-operatively recorded by an anesthesiologist). Gross total resection was defined as no residual nodular contrast enhancement on early post-operative magnetic resonance imaging (MRI) performed within 24–72 h post-surgery. In cases in which no post-operative MRI was available, resection status was stated as “unknown.” For patients undergoing multiple surgeries, only first resection was included. Primary endpoint of the study was PFS, defined as time between first surgery and tumor recurrence or death. Diagnosis of recurrence was based on radiological Response Assessment in Neuro-Oncology (RANO) criteria [25] and patients were screened by MRI for recurrence every three months. Secondary endpoint was OS, defined from the date of first tumor resection to death.

### Statistical methods

All data related to the surgical and anesthetic procedures were obtained from the hospital electronic patient record (i.s.h.med; SAP, Germany). Data relating to deaths were obtained by submitting a batch data request to the residents’ registration office. Study sample size was chosen to include all eligible patients presenting in the 7.5-year period between January 1, 2010, and June 30, 2017; no a priori power analysis was conducted. Patients alive were censored at follow-up closure date (June 30, 2019).

Patients having received TIVA were matched in a 1:2 ratio with those having received INHA. Matching was done without replacement according to known prognostic factors: age (within a tolerance limit of ±5 years), pre-operative KPI, EOR, MGMT-promoter-methylation-status, concomitant and adjuvant chemotherapy, and radiotherapy. Matching process was performed using a macro written for SAS (SAS Institute, Cary, NC). No match was found for two patients who had received TIVA. In 12 cases, only one match was found.

Baseline demographics, prognostic factors and treatment modalities were compared between groups using chi-square and *t-*tests, as appropriate. Cumulative probabilities of survival were plotted using the Kaplan–Meier method. Log-rank test was used to compare the survival times of the groups.

## Results

### Cohort characteristics

From January 1, 2010, to June 30, 2017, a total of 576 adult patients underwent craniotomy for resection of a newly diagnosed, IDH-wildtype GBM under general anesthesia at the Department of Neurosurgery, University Hospital Heidelberg, Germany. After exclusions, 471 were eligible for further analyses. General anesthesia was carried out as INHA in 417 and as TIVA in 54 cases. After matching, 52 patients remained in the TIVA group and 92 patients in INHA group (Fig. 1). All patients in the INHA group received sevoflurane as a volatile anesthetic. For induction of anesthesia, a single dose of propofol was used in all cases. Patient characteristics are summarized in Table 1.

**Table 1:** Demographics and baseline characteristics

	Matched patients			All patients		
	INHA	TIVA	*p* value	INHA	TIVA	*p* value
Variables	(*n* = 92)	(*n* = 52)		(*n* = 417)	(*n* = 54)	
Age (yr)						
Mean (SD)	63 (10.4)	62 (11.4)	0.80^†^	64 (11.4)	62.2 (11.3)	0.39^†^
Gender						
Male (%)	60 (65.2)	33 (63.5)	0.83^††^	252 (60.4)	28 (51.9)	0.23^††^
Female (%)	32 (34.8)	19 (36.5)		165 (39.6)	26 (48.1)	
BMI						
Mean (SD)	25.0 (4.3)	25.5 (4.2)	0.77^†^	26.3 (5.1)	25.5 (4.1)	0.31^†^
Included cases^‡^ (%)	86 (93.5)	47 (90.4)		387 (92.8)	48 (83.3)	
ASA status						
ASA 1&2 (%)	50 (54.3)	33 (63.5)	0.29^††^	242 (58.0)	34 (63.0)	0.48^††^
ASA 3&4 (%)	42 (45.7)	19 (36.5)		175 (42.0)	20 (37.0)	
Karnofsky index						
Mean (SD)	82 (13.3)	82 (12.8)	0.96^†^	81 (14.9)	80.3 (29.3)	0.68^†^
Extent of resection						
Total (%)	22 (23.9)	12 (23.1)	0.92^††^	122 (29.3)	12 (22.2)	0.49^††^
Subtotal (%)	66 (71.7)	37 (71.2)		259 (62.1)	38 (70.4)	
Unknown (%)	4 (4.4)	3 (5.8)		36 (8.4)	4 (7.4)	
MGMT promoter methylation						
Positive (%)	41 (44.6)	24 (46.2)	0.97^††^	147 (35.3)	25 (46.3)	0.06^††^
Negative (%)	41 (44.6)	22 (42.3)		166 (39.8)	23 (42.6)	
Unknown (%)	10 (10.9)	6 (11.5)		104 (24.9)	6 (11.1)	
Radiation therapy						
Yes (%)	72 (78.3)	41 (78.9)	0.93^††^	347 (83.2)	42 (77.8)	0.32^††^
No (%)	20 (21.7)	11 (21.5)		70 (16.79)	12 (22.2)	
Concomitant chemotherapy with temozolomide						
Yes (%)	63 (68.5)	36 (69.2)	0.93^††^	260 (62.4)	37 (68.5)	0.38^††^
No (%)	29 (31.5)	16 (30.8)		157 (37.6)	17 (31.5)	
Adjuvant chemotherapy with temozolomide						
Yes (%)	62 (67.4%)	35 (67.3)	0.99^††^	260 (62.4)	36 (66.7)	0.54^††^
No (%)	30 (32.6%)	17 (32.7)		157 (37.6)	18 (33.3)	
Blood transfusion						
Yes (%)	2 (2.2)	1 (1.9)	0.92^††^	10 (2.4)	1 (1.9)	0.80^††^
No (%)	90 (97.8)	51 (98.1)		407 (97.6)	53 (98.1)	
Time of anesthesia						
Mean (SD) [m]	368 (88)	394 (90)	0.31^†^	380 (106)	395 (93)	0.69^†^

^†^*t*-test; ^††^ chi-square test; ^‡^ data on BMI were incomplete

ASA, American Society of Anesthesiologists; BMI, body mass index; MGMT, O-6-methylguanine-DNA-methyltransferase gene; INHA, volatile inhalational anesthesia; SD, standard deviation; TIVA, total intravenous anesthesia; yr, years.

Mean age was 63 years in the INHA and 62 years in the TIVA group (*p* = 0.80). Likewise, distribution of gender (*p* = 0.83), ASA status (*p* = 0.29), and MGMT-promoter-methylation status (*p* = 0.97) did not differ between groups. Similarly, pre-operative KPI (*p* = 0.96), BMI (*p* = 0.77) and duration of anesthesia did not differ (*p* = 0.31). Only a small fraction of patients in both groups required peri-operative blood transfusion (*p* = 0.92). The vast majority of patients in both groups underwent concomitant chemotherapy including TMZ (INHA: 68.5%; TIVA: 69.2%; *p* = 0.93) and adjuvant radiation therapy (INHA: 78.3%; TIVA: 78.9%; *p* = 0.93). Two-thirds of patients received adjuvant chemotherapy including TMZ (INHA: 67.4%; TIVA: 67.3%; *p* = 0.99).

### Progression-free and overall survival

**Fig. 2.**
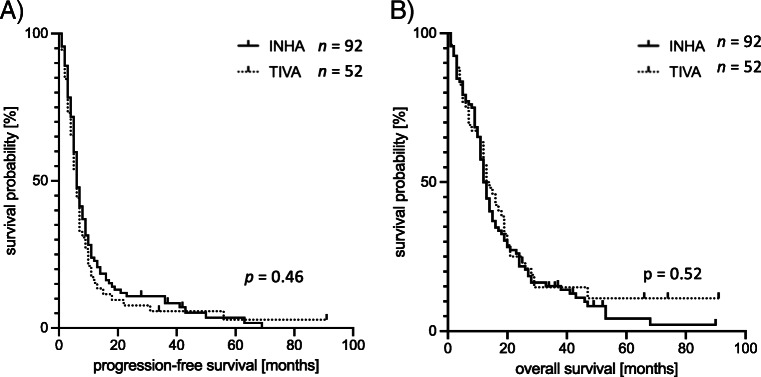
Survival data of matched groups. Kaplan–Meyer plots of progression-free survival a and overall survival **b** from the date of surgery by AT. Survival curves were compared using the log-rank test

Survival data of matched groups and the total cohort are summarized in Table 2. After 24 months of follow-up, 3.3% of patients receiving INHA and 3.8% of patients receiving TIVA during tumor resection survived without progression (*p* = 0.85; matched analysis). Median PFS was 6 months in both groups (*p* = 0.45); 6.5% of patients in the INHA and 13.5% in the TIVA group were alive at the end of follow-up period (*p* = 0.16). Median OS was 13.0 months in the INHA and 13.5 months in the TIVA group (*p* = 0.52). Moreover, there was no difference regarding 1-year PFS (INHA: 22.8% vs. TIVA: 15.4%; *p* = 0.28), 2-year PFS (INHA: 10.9% vs. TIVA: 7.7%; *p* = 0.54), 1-year OS (INHA: 50.0% vs. TIVA: 55.8%; *p* = 0.51), and 2-year OS (INHA: 21.7% vs. TIVA: 25.0%; *p* = 0.65). PFS and OS data are summarized in Table 2. Kaplan–Meier survival curves displaying the probability of survival according to the AT received during tumor resection are shown in Fig. 2.

**Table 2:** Progression-free and overall survival according to group affiliation

	Matched patients			All patients		
	INHA	TIVA	*p* value	INHA	TIVA	*p* value
Variables	(*n* = 92)	(*n* = 52)		(*n* = 417)	(*n* = 54)	
Progression-free survival (PFS)						
n (%)	3 (3.3)	2 (3.8)	0.85^††^	13 (3.1)	2 (3.7)	0.81^††^
Median (min/max) [months]	6 (1/89)	6 (1/91)	0.46^+^	6 (1/89)	6 (1/91)	0.74^+^
One-year PFS						
n (%)	21 (22.8)	8 (15.4)	0.28^††^	77 (18.5)	8 (14.8)	0.51^††^
Two-year PFS						
n (%)	10 (10.9)	4 (7.7)	0.54^††^	31 (7.4)	4 (7.4)	0.99^††^
Overall survival						
n (%)	6 (6.5)	7 (13.5)	0.16^††^	23 (5.5)	7 (13.0)	**0.03**^**††**^
Median (min/max) [months]	13.0	13.5	0.52^+^	13.0 (0/90)	13.5 (1/91)	0.45^+^
One-year survival						
n (%)	46 (50.0)	29 (55.8)	0.51^††^	216 (51.8)	30 (55.6)	0.60^††^
Two-year survival						
n (%)	20 (21.7)	13 (25.0)	0.65^††^	92 (22.1)	13 (24.1)	0.74^††^

^+^Log-rank-Test; ^††^ chi-square test

*INHA* volatile inhalational anesthesia, *TIVA* total intravenous anesthesia

As matching reduced study sample significantly, we asked if there was a difference between patients who received TIVA or INHA with regard to PFS or OS in the total unmatched study cohort (*n* = 471; TIVA_total_ *n* = 54; INHA_total_ *n* = 417); 3.1% of patients in the INHA_total_ group and 3.7% in the TIVA_total_ group remained progression-free until the end of follow-up (*p* = 0.81). Median PFS was 6 months in both groups (*p* = 0.74). Interestingly, the percentage of patients alive at the end of follow-up was significantly lower in the INHA_total_ group (5.5%) than in the TIVA_total_ group (13%; *p* = 0.03). However, median OS did not differ between patients receiving TIVA_total_ (13.5 months) or INHA_total_ (13.0 months; *p* = 0.45). Likewise, neither the percentage of patients surviving the first year post-surgery (INHA_total_ 18.5%, TIVA_total_ 14.8%; *p* = 0.39) nor the percentage surviving the second year post-surgery (INHA_total_: 7.4%, TIVA_total_: 7.4%; *p* = 0.52) differed between groups. In line with this observation, 1-year OS (INHA_total_ 51.8%, TIVA_total_ 55.6%; *p* = 0.82) and 2-year OS (INHA_total_ 22.1%, TIVA_total_ 24.1%; *p* = 0.07) were comparable in both groups.

Moreover, as half of patients receiving TIVA during tumor resection were operated on between January 2015 and June 2017 (Suppl. Fig. 2a), we asked if date of surgery might be a confounder. However, we found no difference regarding PFS of patients operated on between January 2010 and December 2014 and those operated on between January 2015 and June 2017 (Suppl. Fig. 2b; *p* = 0.43). Additionally, as the use of IONM is an indicator of eloquent location, we asked if necessity for IONM might be a bias within the TIVA group towards worse prognosis; 16 of 52 TIVA patients (33.3%) in the matched group were operated on using IONM but there was no difference in survival probability between cases that were operated on using IONM and those where IONM was not used (Suppl. Fig. 2c; *p* = 0.3).

## Discussion

There is increasing evidence that, regardless of the specific tumor entity, the AT used during resection of solid tumors might impact long-term survival of patients, favoring TIVA over INHA [10, 26]. However, due to incomplete data, the largest of those studies did not include staging information of cancer patients, ignoring a crucial possible confounding factor [26]. In the present retrospective study we addressed this question in a more homogenous study sample consisting solely of IDH-wildtype GBM. Comparing patients receiving TIVA and those receiving INHA, we did not find any differences regarding PFS and OS.

Despite of the robust study design, our work has some limitations. First, our cohort contained a limited number of TIVA cases. However, after matching, the compared groups were well balanced with regard to known prognostic factors and even to less important confounding factors, such as gender distribution, comorbidities (reflected by the ASA status), BMI, duration of surgery, or necessity for intraoperative blood transfusion. Of note, also the date of surgery had no influence on either PFS or OS. By matching in a 1:2 ratio, we were able to augment the power of our analysis. Notwithstanding, to reach the level of significance for a difference of about 5% with a p-value of 0.05 and a power of 0.2 (as we found for PFS in our study: 22,8% vs. 15,4%), a prospective study using a 1:1 matching would have to include 2 * 352 = 704 cases [6, 29]. Of note, the observation that the percentage of patients alive at the end of follow-up was significantly lower in the INHA_total_ group than in the TIVA_total_ group (5.5% vs. 13%; *p* = 0.03) can be explained by the fact that half of the patients in the TIVA group were operated in the last two years of the study period. By comparing PFS of these patients to the PFS of those operated earlier, we were able to exclude the date of surgery as a confounder.

Second, we did not explicitly consider tumor volume and the exact location of the tumor related to eloquent brain structures (motor and speech function). However, as we discriminated between total and subtotal resections in the matching process, we probably also differentiated more difficult from easier resections, partially reflecting the proximity of the tumor to structures pivotal for neurological function. By excluding cases in which an open biopsy was taken only to confirm diagnosis before planning a radio-chemotherapy, we excluded cases with contraindications for an extended operation and therefore did not receive maximal therapy. We accepted the resulting bias towards cases with better prognosis, with the intention to keep the patient cohort as homogenous as possible. Given the retrospective nature of our study, it was not possible to deduce the anesthesiologist’s decision for the AT in all cases. In one-third of the TIVA-cases, it was used due to IONM. However, the PFS of TIVA-cases in which IONM was used did not differ from those in which it was not used. Moreover, as patient characteristics (especially EOR as a surrogate for eloquent tumor location) did not differ between the two groups, we consider the potential bias to be negligible.

Third, we cannot exclude that both AT influence outcome similarly. A comparison of patients receiving general anesthesia with those receiving an awake craniotomy for tumor resection might provide insight into this question. However, such an investigation extends beyond the scope of this study. Moreover, all patients included in our analysis received a single dose of propofol (2 mg/kg bodyweight) for anesthesia induction. To our knowledge, there are no data analyzing the effect of such a single injection on solid tumors. However, due to the underlining pharmacokinetics it is not likely: after a single injection, propofol is distributed very quickly from plasma into other compartments (e.g., muscle, fat), causing a fast drop in plasma concentration and in the brain (ending the hypnotic effect within 2–4 min) [19]. After hours of continuous infusion (e.g., during TIVA), a second phase of redistribution from a slow compartment may cause significant plasma levels. However, this effect is negligible after a single dose [19]. All in vitro studies describing an antitumorigenic effect of hypnotic agents used long exposure times of at least several hours at concentrations exceeding those normally achieved during general anesthesia [6–8, 16, 29]. Considering this pharmacological background, it seems unlikely that a single dose of propofol (in the INHA group) before the beginning of the operation has the same effect on tumor cells as an exposure of several hours in higher concentrations (in the TIVA group) during resection of the tumor.

In addition to direct effects on cancer cells, there are two hypotheses seeking to explain the differences in outcome observed in tumor patients receiving either INHA or TIVA during tumor resection. The first hypothesis postulates an increase in natural killer cell activity induced by propofol [2], and the second one emphasizes a detrimental effect of volatile anesthetics suppressing natural killer cell activity and inducing T-lymphocyte apoptosis [8]. However, in a recent prospective in vivo study, Lim et al. did not find any significant differences regarding cancer cell, natural killer cell, or cytotoxic T-lymphocyte function in patients undergoing breast cancer surgery either under TIVA or INHA [11]. In accordance with these findings, our work supports the idea that the impact of narcotic choice on the outcome of GBM patients is, if present, not potent enough to influence PFS or OS. Our results are in line with a recent meta-analysis that could not confirm the hypothesis of an impact of AT on the progression of solid cancers [7]. Although Jin et al. did confirm a lower overall pooled hazard ratio for all-cause mortality in favor of TIVA initially, this finding could not be confirmed in consecutive subgroup analysis of mortality and cancer recurrence in different cancer entities [7]*.*

## Conclusion

Altogether, our work strongly supports the assumption that there is no impact of the anesthesiologist’s choice of hypnotic agent on the outcome of IDH-wildtype GBM patients. However, due to the retrospective nature of the present study, being not able to control possible unknown confounding factors, our work does not replace a prospective randomized controlled trial.

## Supplementary Information

ESM 1 (PDF 227 kb)

## Data Availability

All data is stored for at least 10 years. Extracts are published in the supplements.

## Glossary

**ASA**: American Society of Anesthesiologists
**AT**: anesthetic technique
**EOR**: extent of resection
**GBM**: glioblastoma
**IDH1**: isocitrate-dehydrogenase 1
**INHA**: inhalative anesthesia
**IONM**: intraoperative neurophysiological monitoring
**KPI**: Karnofsky performance index
**MGMT**: O-6-methylguanine-DNA-methyltransferase
**MRI**: magnetic resonance imaging
**OS**: overall survival
**PFS**: progression-free survival
**RANO**: Response Assessment in Neuro-Oncology
**TIVA**: intravenous anesthesia
**TMZ**: temozolomide
**WHO**: World Health Organization
