# Supplementary material for: The anesthetist’s choice of inhalational vs. intravenous anesthetics has no impact on survival of glioblastoma patients
Source: Neurosurg Rev. 2020 Dec 22;44(5):2707–15. doi: 10.1007/s10143-020-01452-7 (PMC8490243; doi:10.1007/s10143-020-01452-7)
Supplement: Supplementary file 1 — (PDF 227 kb) [file 10143_2020_1452_MOESM1_ESM.pdf]

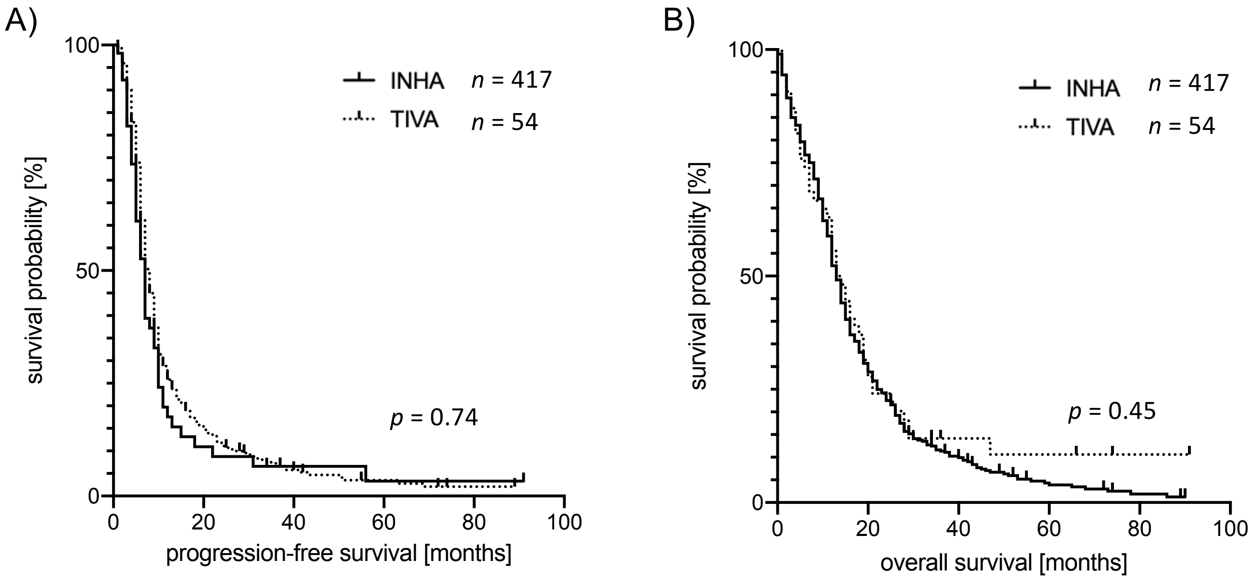

Suppl. Fig. 1. Data of all patients included. Kaplan–Meyer plots of progression-free survival (A) and overall survival (B) from the date of surgery by anesthetic technique. Survival curves were compared using the log-rank test.

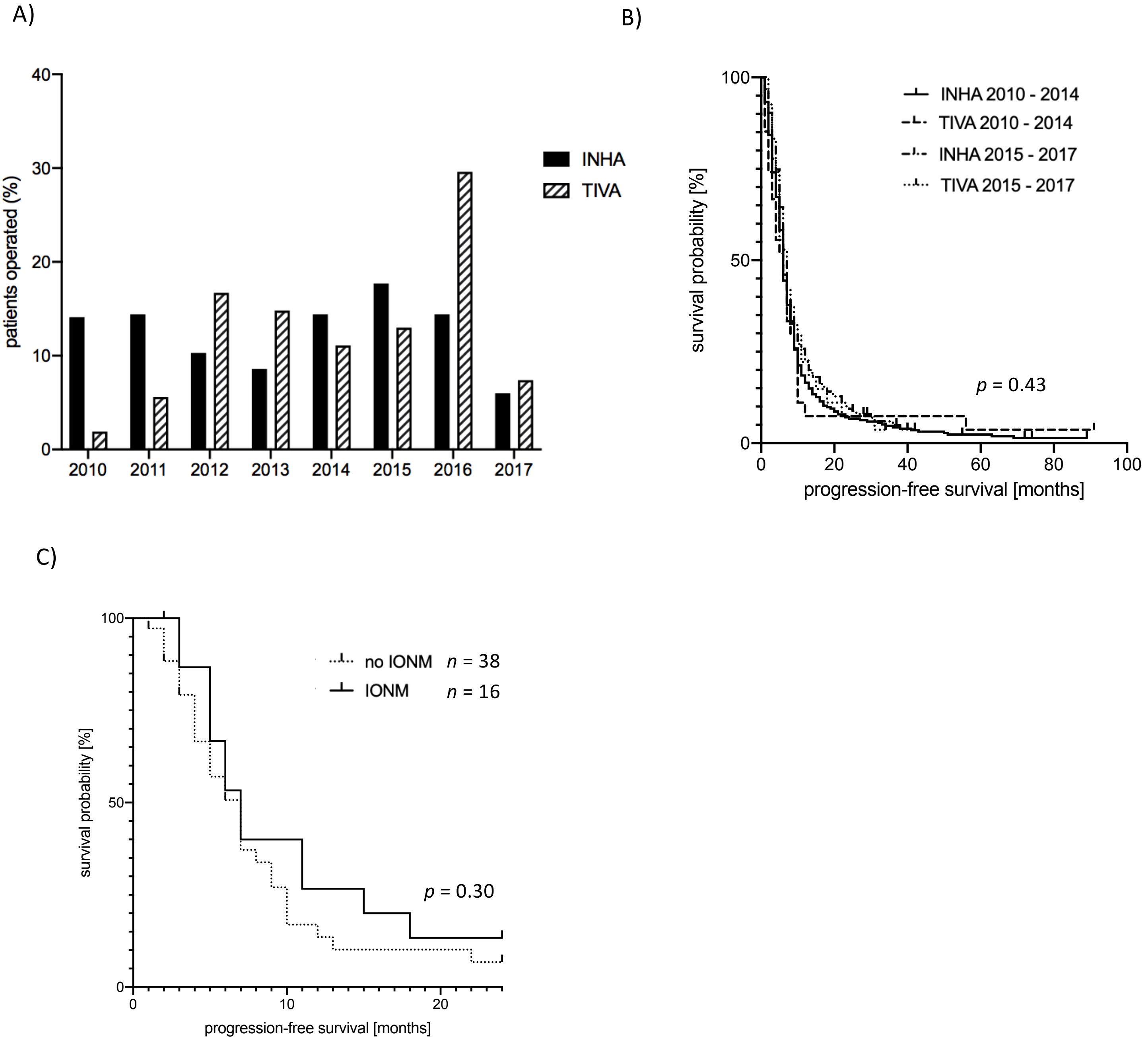

Suppl. Fig. 2. (A) Percentage of patients operated on according to year and group (INHA, n = 417; TIVA, n = 54). (B) Kaplan–Meyer plots of progression-free survival (PFS) of patients operated on during 2010–2014 and 2015–2017 according to anesthetic technique during tumor resection. (C) Kaplan–Meyer plots of PFS of patients up to 24 months post-surgery who received TIVA and were operated on with and without intraoperative neurophysiological monitoring.
